# Social Capital and Depressive Episodes: Gender Differences in the ELSA-Brasil Cohort

**DOI:** 10.3389/fpubh.2021.657700

**Published:** 2021-05-17

**Authors:** Ester Paiva Souto, Arlinda B. Moreno, Dóra Chor, Enirtes C. Prates Melo, Sandhi M. Barreto, Maria Angélica Nunes, Rosane Harter Griep

**Affiliations:** ^1^Department of Epidemiology and Quantitative Methods in Health, National School of Public Health, Oswaldo Cruz Foundation, Rio de Janeiro, Brazil; ^2^Postgraduate Program in Epidemiology, Faculty of Medicine, Universidade Federal do Rio Grande do Sul, Porto Alegre, Brazil; ^3^Laboratory of Health and Environment Education, Oswaldo Cruz Institute, Oswaldo Cruz Foundation, Rio de Janeiro, Brazil

**Keywords:** depression, social capital, gender difference, Brazil, cohort studies, multinomial regression

## Abstract

**Introduction:** The association between social capital and depression is a frequent research topic in developed countries, often with inconclusive results. Furthermore, for both social capital and depression, there are gender differences established in the literature. This study investigates gender differences in the association of social capital with the incidence and maintenance of depressive episodes.

**Methods:** Baseline and second wave data (4 years of follow-up) from the Longitudinal Study of Adult Health (ELSA-Brasil), a multicenter cohort of civil servants with 15,105 workers aged 35–74 years, were used. Social capital was assessed using the Resource Generator, a scale composed of two different dimensions: “social support” and “prestige and education.” Depressive episodes were assessed using the Clinical Interview Schedule - Revised (CIS-R). The statistical analysis was performed using multinomial regression with adjustments for possible confounding factors.

**Results:** Among men, low social capital in the “social support” dimension was associated with the incidence of depressive episodes (RR = 1.66; 95% CI: 1.01–2.72). Among women, social support was associated with the maintenance of depressive episodes (RR = 2.66; 95% CI: 1.61–4.41). Social capital was not associated with the incidence or maintenance of depressive episodes in the “prestige and education” dimension in both genders.

**Conclusion:** The results highlight the importance of the dimension “social support” in both genders in its association with mental health. The resource-based social capital approach proved to be adequate for investigating mental health and confirms the idea that social networks can be useful in the treatment and prevention of depressive episodes.

## Introduction

It is estimated that, among mental disorders, depression should be the main cause of health problems and premature death in the world in 2030 (1). It is a multifactorial and heterogeneous disease, which has a major impact on mental health, affecting more than 300 million people globally (its prevalence was estimated at 4.4%). Currently depression is one of the main risk factors for suicide and the biggest contributor to YLD (years lived with disability), corresponding to 7.5% of all years lived with disability in 2015 (2).

Different risk factors are attributed to the incidence or recurrence of depression. While sociodemographic characteristics such as gender, marital status and socioeconomic status seem to be linked to the incidence, recurrence is more associated with factors related to vulnerability to depression, such as family history of depression and the presence of certain symptoms or their severity in the first depressive episode (3).

Investigations on how social capital can be an important factor in understanding the emergence of depression have been increasingly frequent in recent years (4–7). Social capital is a multidimensional construct focused on the presence of resources that individuals or groups can access through a network of relationships. This construct discusses the role of social networks to access these embedded resources, and it has been investigated concerning various outcomes in mental health, like common mental disorders, depression, child mental illness (8). Its elements are presented by several authors who approach it in an interconnected way, describing it as social trust, social support, reciprocity, civic engagement and social control (9–11).

Aware of the importance of social networks in maintaining psychological well-being (12), the main theories that seek to elucidate the mechanisms by which specific aspects of social networks lead to the maintenance or improvement of psychological well-being state that they can act directly (through social control) or act as a protection mechanism against stress (13). In the conception developed by Bourdieu (9), social capital refers to social resources made available through networks that help the individual to obtain benefits.

An expanded view of social capital, provided by Putnam (11), places it as group property, developed and enjoyed in a community collectively. However, we can understand that the basis for generating individual or collective resources are social networks characterized by social trust (14) and that the investigation of the availability of resources can translate the importance of social capital for individuals and/or for the society.

Theoretical advances in social capital research also classify it as having cognitive components (perceptions of social support and trust) and structural components (related to the structure of the social network) (15). Cognitive social capital is more often linked to mental health outcomes (16) compared to structural social capital. It is also known that the association between social capital measured only at the collective level and mental health has less explored than the association with social capital at the individual level (8).

Studies that investigate the presence of social capital linked to resources available on networks, also find associations with positive effects on mental health and depression. The association between less access to social capital resources and the presence of common mental disorders has been identified in the United Kingdom (17). In parallel, the association between low levels of social capital and depressive traits was identified in a study with women from Southeast Asia (18).

A model of vulnerability to stress can be used to link the way that social capital protects against the incidence or relapse of a depressive episode. These models postulate that individual vulnerabilities interact with adverse life events or stressors to cause a certain pathology (19). Vulnerability models that explore the interaction between genetics and the environment for the development of depression are frequent (20, 21). Similarly, an extension to the vulnerability model developed by Harris (22) considers the psychosocial aspects in the development of depression, suggesting that the presence of social capital made available through social networks can influence its course. In this model, the absence of social capital would constitute an individual vulnerability capable of promoting the appearance of depression or its maintenance.

Thus, understanding the importance of social networks in investigating the occurrence and course of depression, this study seeks to examine the association of different dimensions of social capital, in the incidence and maintenance of depressive episodes in a large Brazilian cohort of workers. The need and importance of studying social capital and health longitudinally has been indicated in the international literature (23, 24). This is the first Brazilian study to investigate gender differences in the association between social capital and depressive episodes in a prospective manner and similar to existing cross-sectional studies (17, 18, 25), and its main hypothesis is that a higher level of social capital is inversely associated with the occurrence and maintenance of depressive episodes and that this association is differentiated according to the gender of the participants.

## Materials and Methods

### Study Design, Population and Data Collection

The Longitudinal Study of Adult Health (ELSA-Brasil) is a prospective and multicenter cohort study, with a baseline composed of 15,105 civil servants from six Brazilian teaching and research institutions (FIOCRUZ, UFBA, UFES, UFMG, UFRGS and USP), aged between 35 and 74 years old. This study was primarily designed to investigate the incidence and progression of cardiovascular diseases and diabetes and its social, occupational, environmental, psychological and biological determinants, which gives it a multidisciplinary character.

**Figure 1:**
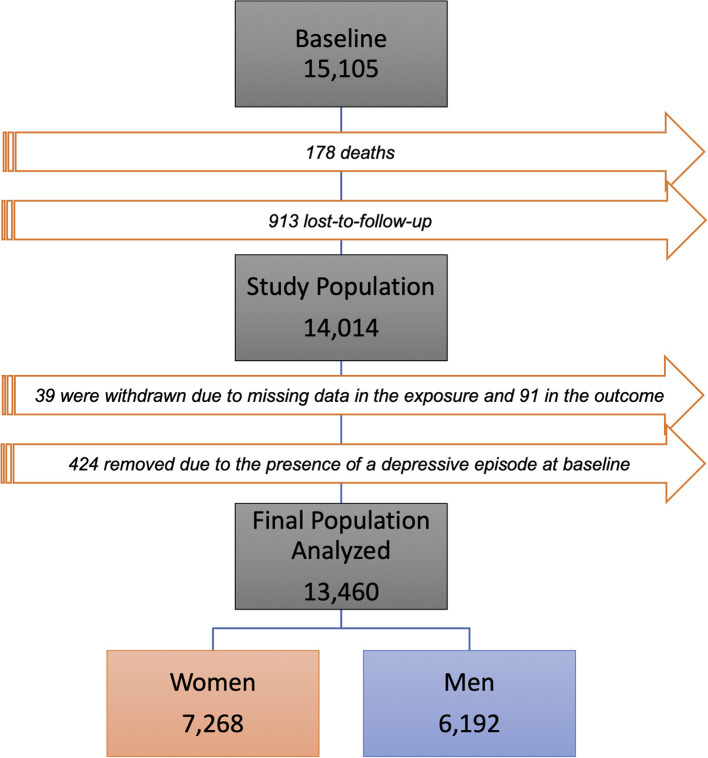
Flowchart of the composition of the study population.

Baseline data collection was carried out between 2008 and 2010, consisting of interviews and exams applied by trained and certified staff. As planned, a second wave of exams and interviews was carried out between 2012 and 2014. In addition, the monitoring of outcomes has been carried out through annual telephone contacts, at which time information about hospitalizations, new diagnoses of diseases and deaths is collected. More detailed descriptions of the methodological aspects of ELSA-Brasil can be found in Schmidt et al. (26). As illustrated in Figure 1, to compose the study population, from the participants in the first follow-up, those who failed to answer any of the social capital items, or the questions that make up the depressive episode classification in the CIS-R (Clinical Interview Schedule - Revised), were excluded in both waves. No participant presented missing data in the adjustment variables. We also excluded participants who had a depressive episode only at the baseline. Thus, 554 people were excluded, resulting in an analyzed population of 13,460 participants.

ELSA-Brasil had its protocols submitted and approved by the Research Ethics Committees of the Oswaldo Cruz Foundation (FIOCRUZ) and the National Commission for Research Ethics (CONEP). This project was submitted and approved by the Institution's Ethics Committee (CEP 02392412.0.0000.5240).

### Study Variables

Social Capital - the Brazilian version of the Resource Generator (RG) was used (27). Such a scale is considered a simple and objective instrument to quantify access to the resources available on social networks. Elaborated in the tradition of network analysis, this scale captures structural data (quantity and source of resources) directly and allows inferring cognitive aspects as it incorporates the concepts of trust and reciprocity in social networks. The original version (28) consists of 33 items that aim to evaluate different dimensions of Social Capital. The questions, operationalized in one or two stages, present some everyday situations to discriminate the reach to different types of resources. The individual answers whether or not they have access to a particular resource and soon after, if the answer is positive, they specify the source of the resource (family, friends or acquaintances).

The Brazilian version underwent reliability and validity studies that assessed the adequacy of the translation and items and their dimensional structure (27, 29). Thus, the scale used in this research follows the structure proposed in the validity study, which considers only two dimensions of the scale: “prestige and education” (13 items) and “social support” (10 items). In addition, we consider only the presence of the resource; and not its source.

In each dimension, social capital was classified as low, medium and high, dividing the score approximately into terciles and thus the dimension of prestige and education had scores between 0 and 9, 10 and 12, and 13 points and in the dimension of social support, the scores were between 0 and 7, 8 and 9, and 10 points, respectively. The social capital scale was applied only at the baseline, considering that the level of social capital is a stable variable, which does not show significant variations in the short time between the two measures (4 years).

#### Depression

The presence of depression in the last 7 days was investigated using a Brazilian Portuguese version of the CIS-R (30). The CIS-R is a structured psychiatric interview, relatively brief and suitable for lay interviewers. It was applied in face-to-face interviews and the presence of depressive episodes was determined by algorithms based on the ICD-10 (international classification of diseases−10th revision) diagnostic criteria for depressive episodes (group F32.xx) without psychotic symptoms. Introductory questions from the CIS-R that consider appetite and weight variation were not used, which may underestimate the prevalence of depressive episodes.

In the two waves of data collection, depressive episodes [mild, moderate (with or without somatic symptoms) and severe] were grouped into a dichotomous variable such as the presence or absence of a depressive episode. For this study, these dichotomous variables present in the two waves make up a third variable with three categories, as follows:

The presence of a depressive episode at baseline and in the first follow-up was called “maintenance of depression” (value = 1). The “incidence” (value = 2) corresponded to the presence of a depressive episode only in the first follow-up, while the category “not depressed” (value = 3), used as a reference, was named for the participants who were not classified for depressive episodes in either wave. Participants who had depressive episodes only at the baseline were excluded.

#### Covariables

Age (continuous), marital status (single, separated/divorced, married/united and widowed) and education (complete elementary school, complete high school and complete higher education/postgraduate).

### Data Analysis

Gender differences in associations previously investigated in the literature (6, 31) motivated the stratification by gender in the analyzes. The distribution of confounding variables and the variation of social capital at three levels in the two dimensions of social capital were assessed through comparisons using the chi-square test for categorical variables. This analysis was performed at a 5% level of significance. For the selection of variables that were inserted in the multivariate analysis, the level of significance was 10%.

A series of multinomial regression models were implemented to separately assess the association between the two dimensions of social capital and depressive symptoms. The Relative Risk (RR) and respective 95% confidence intervals (95% CI) were estimated, considering the three categories of the outcome (not depressed, incidence and maintenance). The multinomial models (the two dimensions of social capital with depression) suffered sequential adjustments from the gross estimate (Model 0), as follows: inclusion of age (Model 1), education (Model 2) and marital status (Model 3). The variables maintained in the final models were those that remained associated with the response variables at a significance level of 5%. The analysis was performed using the R program (v.3.4.1).

## Results

We observed a higher proportion of women (54.0%), with a similar average age (52 ± 9 years) for men and women. More than half of men (50.5%) and women (55.7%) had at least a complete higher education level. 82.2% of men and 55.1% of women were married (Table 1). The incidence of depression was 2.4 and 4.8% and maintenance was 0.5 and 1.8%, respectively, in men and women.

**Table 1:** Characterization of men and women according to the level of social capital of ELSA-Brasil.

**Variables**	**Categories**	***n* total (%)**	**Depression**		
			**Not depressed**	**Incidence**	**Maintenance**
			**(%)**	**(%)**	**(%)**
		**MEN** ***n*** **=** **6,192**			
Prestige and Education	Low	1,115 (18.0)	96.7	2.6	0.7
	Average	1,988 (32.1)	97.7	2.0	0.4
	High	3,089 (49.9)	97.1	2.5	0.4
Social Support	Low	669 (10.8)	95.5	3.6	0.9
	Average	1,684 (27.2)	97.4	2.1	0.5
	High	3,839 (62.0)	97.4	2.3	0.4
Age	35–44	1,448 (23.4)	96.5	3.1	0.4^*^
	45–54	2,446 (39.5)	97.0	2.6	0.4
	55–64	1,639 (26.5)	97.7	1.8	0.5
	65–74	659 (10.6)	98.0	1.4	0.6
Education	Complete elementary school	917 (14.8)	96.4	2.8	0.8^*^
	Complete high school	1,972 (31.8)	96.4	2.7	0.8
	Complete higher education/Postgraduate	3,129 (50.5)	97.9	1.9	0.2
Marital status	Single	322 (5.2)	97.2	2.5	0.3^*^
	Divorced	713 (11.5)	96.1	3.1	0.8
	Married/united	5,089 (82.2)	95.9	2.2	0.4
	Widowed	68 (1.1)	97.4	2.9	1.5
		**WOMEN** ***n*** **=** **7,268**			
Prestige and Education	Low	1,125 (15.5)	92.4	5.3	2.8^*^
	Average	2,546 (35.0)	93.8	4.2	1.9
	High	3,597 (49.5)	93.3	4.9	1.4
Social Support	Low	684 (9.4)	91.5	5.0	3.5^*^
	Average	1,975 (27.2)	92.8	4.7	2.5
	High	4,609 (63.4)	93.8	4.9	1.3
Age	35–44	1,614 (22.2)	93.3	4.6	2.0^*^
	45–54	2,908 (40.0)	92.3	5.5	2.2
	55–64	2,081 (28.6)	94.0	4.8	1.2
	65–74	665 (9.2)	96.1	2.6	1.4
Education	Complete elementary school	627 (8.6)	90.3	6.4	3.3^*^
	Complete high school	2,594 (35.7)	91.5	5.9	2.5
	Complete higher education/Postgraduate	4,047 (55.7)	95.0	3.9	1.1
Marital status	Single	1,065 (14.7)	95.4	3.3	1.2^*^
	Divorced	1,940 (26.7)	92.0	5.4	2.6
	Married/united	4,008 (55.1)	93.6	4.9	1.6
	Widow	467 (6.4)	92.1	6.1	1.7

* *p-value < 0.05*.

The distribution of social capital was similar between the genders, with higher frequencies of availability of high social capital being observed for both. For the “prestige and education” dimension, the frequencies of low, medium and high social capital for men were 18.7, 32.1, and 49% and among women 16.4, 35.0, and 48, 6%. For the “social support” dimension, 11.4, 27.7, and 60.9% for men and 10.2, 27.4, and 62.4% for women, respectively.

It has also been observed that the incidence of depression decreases and maintenance increases slightly among older men. In women, the age group with the highest incidence and maintenance of depression is between 45 and 54 years old. Only among men there was no association between the dimensions of social capital and the presence of depressive episodes (Table 1).

Among men, we found no association between having low social capital in both dimensions and maintaining depressive episodes, when compared to men with high social capital. However, in the dimension of social support, after adjustments by the variables, men with low social capital have a 66% higher incidence of depression than those with high social capital (Table 2).

**Table 2:** Association between social capital, incidence and maintenance of depression among men from ELSA-Brasil.

	**MEN (*****n*** **=** **6,192)**			
Social capital^*^	**Prestige and education (RR)**		**Social support (RR)**	
**Ref. not dep. (*n* = 6,020)**	**Incidence (*n* = 142)**	**Maintenance (*n* = 28)**	**Incidence (*n* = 142)**	**Maintenance (*n* = 28)**
**Model 0**^**a**^				
Average CS	0.73 (0.49–1.09)	0.83 (0.33–2.09)	0.90 (0.60–1.34)	1.30 (0.54–3.10)
Low CS	0.96 (0.62–1.49)	1.71 (0.70–4.13)	1.51 (0.94–2.43)	2.53 (0.97–6.61)
**Model 1**^**b**^				
Average CS	0.75 (0.51–1.12)	0.82 (0.32–2.05)	0.98 (0.65–1.47)	1.26 (0.52–3.01)
Low CS	1.04 (0.67–1.63)	1.64 (0.68–3.99)	**1.81 (1.12–2.95)**	2.37 (0.89–6.31)
**Model 2**^**c**^				
Average CS	0.71 (0.47–1.06)	0.59 (0.23–1.50)	0.94 (0.62–1.41)	1.00 (0.41–2.41)
Low CS	0.87 (0.54–1.40)	0.78 (0.30–1.99)	**1.68 (1.03–2.76)**	1.60 (0.59–4.33)
**Model 3**^**d**^				
Average CS	0.71 (0.48–1.06)	0.60 (0.24–1.54)	0.93 (0.62–1.40)	0.98 (0.41–2.38)
Low CS	0.87 (0.54–1.41)	0.78 (0.30–2.00)	**1.66 (1.01–2.72)**	1.56 (0.58–4.24)

a *Gross Model*.

b *Age-adjusted model*.

c *Model 1 + education*.

d *Model 2 + marital status*.

* *Reference Category: high. Significant results in bold*.

Among women, we did not observe significant associations in the final model in the dimension “prestige and education” in the incidence and maintenance of depressive episodes. However, low social capital showed a significant association with the maintenance of depressive episodes before the inclusion of the covariable education level [Model 1: RR = 2.10 (1.33–3.31)]. The other adjustments decrease the strength of the association and the confidence interval makes it non-significant (Table 3).

**Table 3:** Association between social capital and the incidence and maintenance of depression among women from ELSA-Brasil.

	**WOMEN (n** **=** **7,268)**			
Social capital^*^	**Prestige and education (RR)**		**Social support (RR)**	
**Ref. not dep. (*n* = 6,793)**	**Incidence (*n* = 344)**	**Maintenance (*n* = 131)**	**Incidence (*n* = 344)**	**Maintenance (*n* = 131)**
**Model 0**^**a**^				
Average CS	0.82 (0.65–1.05)	1.35 (0.91–2.01)	0.97 (0.75–1.25)	**2.08 (1.42–3.05)**
Low CS	0.96 (0.70–1.31)	**1.98 (1.26–3.11)**	1.08 (0.75–1.57)	**2.95 (1.82–4.79)**
**Model 1**^**b**^				
Average CS	0.83 (0.65–1.06)	1.37 (0.92–2.04)	1.01 (0.78–1.29)	**2.23 (1.52–3.29)**
Low CS	1.00 (0.73–1.36)	**2.10 (1.33–3.31)**	1.17 (0.80–1.70)	**3.41 (2.09–5.59)**
**Model 2**^**c**^				
Average CS	0.75 (0.59–0.97)	1.17 (0.78–1.75)	0.91 (0.71–1.18)	**1.94 (1.31–2.86)**
Low CS	0.77 (0.55–1.07)	1.40 (0.87–2.27)	1.00 (0.68–1.46)	**2.68 (1.62–4.45)**
**Model 3**^**d**^				
Average CS	0.75 (0.58–0.96)	1.16 (0.78–1.74)	0.91 (0.70–1.18)	**1.92 (1.30–2.85)**
Low CS	0.76 (0.54–1.06)	1.38 (0.85–2.23)	1.00 (0.68–1.47)	**2.66 (1.61–4.41)**

a *Raw Model*.

b *Age-adjusted model*.

c *Model 1 + education*.

d *Model 2 + marital status*.

* *Reference Category: high. Significant results in bold*.

Similar to the “prestige and education” dimension, among women, in the “social support” dimension, social capital was not associated with the incidence of depression. However, it was observed that in the final model, compared to women with high social capital, those with low social capital have a 166% higher risk of maintaining depressive episodes (Table 3).

## Discussion

Research with the objective of explaining the association between social capital and depression, although frequent, is conducted mainly in developed countries with inconclusive results. In addition, although differences between the genders regarding social capital and depression are established in the literature, few studies consider and investigate these differences. The aim of this study was to examine the association of social capital, the incidence and maintenance of depressive episodes in a cohort of workers. This is possibly the most extensive investigation ever carried out in Brazil on the influence of social capital in depressive episodes, and the study of the differences between men and women found in this association.

The models showed different results between genders. Among women, an association between the dimension of “social support” and the maintenance of depressive episodes was identified. Among men, low “social support” was associated with the incidence of depressive episodes. These findings helped to clarify how social capital works in the course of depression and has differences related to gender. Additionally, the results showed that not all elements of the social capital evaluated are associated with depressive episodes, since the associations were restricted to the “social support” dimension.

As in our design, some studies also chose to explore the differences between the genders in the level of social capital with outcomes in mental health and chose to stratify the population, finding more frequent associations among women (6, 31, 32). In addition, the social capital measured by network analysis has different characteristics between men and women, which can explain the strongest associations found in our study. Women have more extensive social networks (33) and higher levels of social participation (34), they maintain a greater number of intimate relationships than men, while they depend more on their financial resources. Compared to men, women mobilize more social support during periods of stress and tend to be more vulnerable when they face negative life events and do not have social support (35).

Despite the increase in scientific production on the topic of social capital, the diversity of instruments and approaches in which we can measure it makes it difficult to understand whether opposite results are real divergences or due to methodological variability. With the measure of depression, the difficulty is similar. A study carried out with elderly people in Mexico, which measured cognitive aspects of individual social capital (perception of support, reciprocity and trust), found a higher incidence (36) of depressive episodes (measured with the geriatric depression scale) among women with low social capital. In our study, women with low social capital, compared to those with high level, did not show significant differences related to the incidence of depressive episodes in the two dimensions of social capital. This result, although not expected, can be explanatory about the differences in risk factors associated with the incidence of depression. Considering that the presence of a depressive episode increases the chances of a new episode occurring, perhaps for women, the lack of social capital is more relevant in the recurrence, since support networks do not influence the first episode, but the absence of them can be decisive for a new episode, since different factors are related to the incidence or recurrence of depression (3).

Among men, the importance of capital appeared in the incidence of depressive episodes among those who had low social capital compared to those with high social capital. We believe that the maintenance of depressive episodes is also influenced by the level of social capital, however, we have no statistical power to demonstrate. The small number of men who were classified in the category of maintenance of episodes in our study (*n* = 28), makes clearer conclusions about the role of social capital in this association difficult.

This is the first Brazilian longitudinal study that seeks to assess the influence, on mental health, of resource-based social capital using the Resource Generator, which captures a list of useful resources capable of reflecting different aspects of social capital (28). Studies that use this scale are still infrequent, since its conception is recent. However, valid results have been found in its application regarding problems in mental health in several countries (17, 31, 37).

The two dimensions of the scale seem to relate differently to mental health, corroborating the multidimensionality of social capital. Resources such as “prestige and education” may not be useful for mental health, unlike the dimension of “social support,” which reflects the support given in daily activities and emotional support, such as counseling and information exchange. Similar results were found in a Chinese study that used RG among rural workers to investigate its association with mental health (measured through a dimension of the Short Form Health Survey-36). Stronger associations were found among women in the Domestic Resource and Problem Solving dimensions (which in the Brazilian adaptation corresponds to issues related to “social support”) (31).

We found many studies indicating the importance of social support when associated with mental health (38, 39). The fact that only the dimension of social support was related to depressive episodes, leads us to believe that in these surveys, social capital is not measuring the same as social support. In addition, both the definition and the way to measure social support may vary among theorists (39). Thus, we understand that there may be an overlap of these two constructs, although previous studies indicate that both the conceptualization and the causal paths by which social capital and social support affect health are different (13, 40).

We can also highlight some methodological implications in the use of the Resource Generator. Network analysis scales such as Position Generator (PG) and Name Generator (NG), are referred to as scales that investigate structural social capital, and Resource Generator, despite coming from the theoretical tradition of these scales, can also be indicative of capital cognitive social, given that it captures aspects such as social trust and reciprocity when questioning the expectation of access to resources.

We also know that studies that used PG and NG (41, 42), point out their limitations when indirectly measuring social capital resources, reporting that these scales can underestimate the quantity and quality of mental health-related resources embedded in social networks. Thus, we believe that RG is an appropriate tool, available for resource-based analyzes and that its use in future studies is a good strategy to corroborate the results found.

For this study, the measurement of social capital was performed only at the baseline, and, similar to previous studies (36), it was considered that the level of social capital would remain stable in the follow-up. However, we understand that this may be a limitation in the study design. Furthermore, after an extensive literature review on the factors associated with social capital and depression, we chose to consider age, marital status, and education as covariables that may affect the association between social capital and mental health. Nevertheless, possible residual confounding may be an additional limitation of the study.

We must also consider a certain weakness in the categorization of our outcome, because although the CIS-R is an excellent tool based on the ICD-10, it refers to the last 7 days, so that our conclusions refer to a depressive episode and not to depression throughout life. Furthermore, the use of just two points in time has limited our ability to capture the fluctuations in the course of depression that many individuals experience. Thus, what we can affirm is that our reference category represents a greater probability of presenting individuals with no history of depressive episodes, that the incidence represents someone who has had at least one episode and that the maintenance category represents a greater probability of a recurrent condition. The exclusion of participants who had a depressive episode only at the baseline to focus on considering the association of social capital with depression incidence and recurring depression condition is also a potential limitation of this categorization.

Despite the limitations presented, our study was permeated by important characteristics that confer quality in all its stages. Our data, from the ELSA-Brasil cohort, were collected with strict quality control, outlined longitudinally, allowing inferences of causality, in addition to being collected in a multicenter way, with representation from several Brazilian cities. All the quality present in the planning and execution enabled great adherence and adherence to the study, given that the losses from follow-up were low (7.2%).

We know that the predictive value of social capital and its scope are not consensual and that longitudinal studies between social capital and depression are still scarce. In this way, this study helps to signal the need for longitudinal investigations that invest in valid and multidimensional instruments to capture the specific mechanisms by which social capital is associated with depression. The findings of our study, recognizing the differences in the course of depression related to gender and the different dimensions of social capital, constitute an important step in the identification of these mechanisms. They not only provide necessary information to guide intervention planning, but also help to identify the need to invest in increasing social capital, as a way to minimize the burden caused by depression in the Brazilian population.

## Data Availability Statement

The datasets presented in this article are not readily available because the ELSA study has government funding and the database is available only to researchers and students of the research institutions linked to the study. Requests to access the datasets should be directed to elsa@fiocruz.br.

## Ethics Statement

The studies involving human participants were reviewed and approved by the research ethics committees of all six centers (Federal University of Minas Gerais—UFMG: 186/06; São Paulo University—USP: 669/06; Federal University of Rio Grande do Sul—UFRGS: 194/061; Federal University of Espírito Santo—UFES: 041/06; Federal University of Bahia—UFBA: 027/06; Oswaldo Cruz Foundation—FIOCRUZ: 343/06). The patients/participants provided their written informed consent to participate in this study.

## Author Contributions

ES, AM, and RG: Conceptualization, Methodology, and Formal analysis. RG and DC: Resources. ES: Writing-original draft preparation. ES, AM, DC, MN, RG, EM, and SB: Writing-review and editing. All authors have read and agreed to the published version of the manuscript.

## Conflict of Interest

The authors declare that the research was conducted in the absence of any commercial or financial relationships that could be construed as a potential conflict of interest.
